# 255. Clinical, echocardiographic and electrocardiographic manifestations of COVID-19 multisystem inflammatory syndrome in children in the Dominican Republic

**DOI:** 10.1093/ofid/ofac492.333

**Published:** 2022-12-15

**Authors:** Yori A Roque, David De Luna, Alfredo J Mena Lora, Kelvin Espinal, Franzory Peralta, Hilario Maria

**Affiliations:** Hospital Metropolitano de Santiago (HOMS), Santiago, Santiago, Dominican Republic; Hospital Metropolitano de Santiago, Santiago, Santiago, Dominican Republic; University of Illinois Chicago, Chicago, Illinois; Hospital Infantil Regional Universitario Dr. Arturo Grullón, Santiago, Santiago, Dominican Republic; Hospital Infantil Regional Universitario Dr. Arturo Grullón, Santiago, Santiago, Dominican Republic; Hospital Infantil Regional Universitario Dr. Arturo Grullón, Santiago, Santiago, Dominican Republic

## Abstract

**Background:**

The COVID-19 pandemic has spread globally and millions of infections have occurred. As cases mount, atypical manifestations of COVID-19 and post-infectious complications such as multisystem inflammatory syndrome in children (MIS-C) become more likely. MIS-C is a life threatening post-infectious complication of COVID-19. There is a paucity of data of MIS-C in the Dominican Republic (DR). We seek to understand the clinical manifestations of MISC-C in the DR.

**Methods:**

This is a retrospective review of cases admitted to a pediatric hospital in the Dominican Republic from March 2020 to December 2021. Patients with clinical findings and a diagnosis of MIS-C were included. Echocardiographic (Echo) and electrocardiographic (ECG) changes were reviewed.

**Results:**

A total of 16 patients were included in our study, of which 68.75 were male. Ages were 12.5% < 1 years old, 12.5% between 1-4, 62.5% 5-12 and 12.5% over 12. Fever and rash were the most common clinical findings (Figure 1), while 69% had a new abnormality on echo and 50% had new ECG abnormalities. Echocardiographic findings are listed in Figure 2.

Clinical findings in patients admitted with MIS-C

**Figure:**
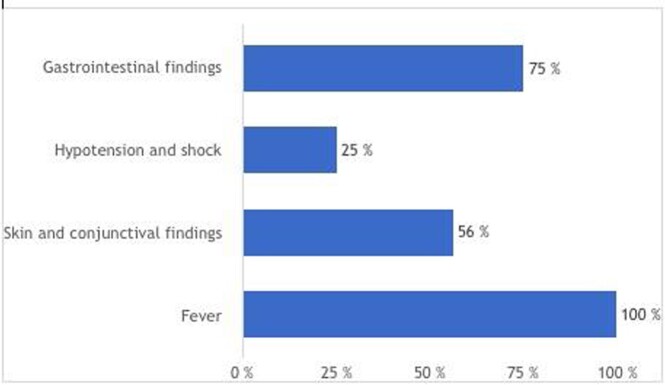

Echo findings

**Figure:**
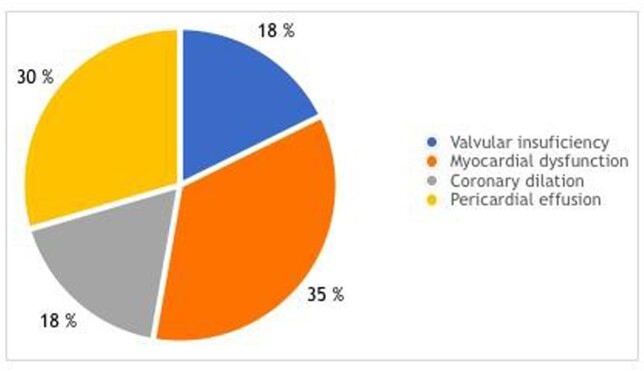

ECG findings

**Figure:**
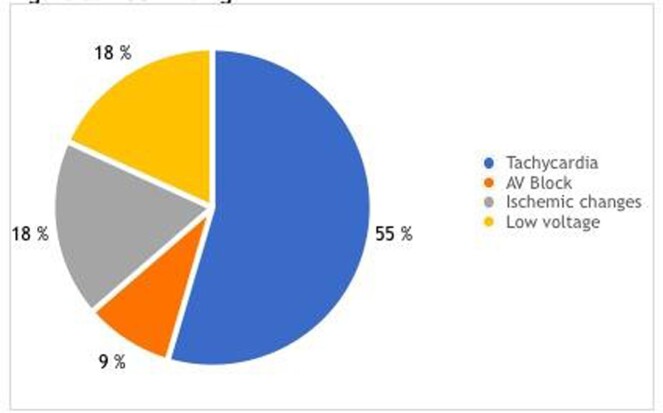

**Conclusion:**

The clinical manifestation of MIS-C are primarily fever, conjunctivitis, rash and hypotension. Because these findings can be non-specific, a high level of suspicion is needed. With over two thirds of patients with MIS-C showing echocardiographic changes and more than 50% showing ECG changes, these two tests can add significant diagnostic value in the right clinical setting. Clinicians should consider early echocardiography and ECG in patients with possible or suspected MIS-C.

**Disclosures:**

**All Authors**: No reported disclosures.

